# High-Risk Oncogenic Human Cytomegalovirus

**DOI:** 10.3390/v14112462

**Published:** 2022-11-07

**Authors:** Georges Herbein

**Affiliations:** 1 Department Pathogens & Inflammation-EPILAB EA4266, University of Franche-Comté UBFC, 25000 Besançon, France; georges.herbein@univ-fcomte.fr; Tel.: +33-381-665-616; 2 Department of Virology, CHRU Besançon, 25000 Besançon, France

**Keywords:** HCMV, high-risk, low-risk, HR-HCMV, LR-HCMV, cancer, oncomodulation, oncogenesis, PGCC, polyploid giant cancer cells

## Abstract

Human cytomegalovirus (HCMV) is a herpesvirus that infects between 40% and 95% of the population worldwide, usually without symptoms. The host immune response keeps the virus in a latent stage, although HCMV can reactivate in an inflammatory context, which could result in sequential lytic/latent viral cycles during the lifetime and thereby participate in HCMV genomic diversity in humans. The high level of HCMV intra-host genomic variability could participate in the oncomodulatory role of HCMV where the virus will favor the development and spread of cancerous cells. Recently, an oncogenic role of HCMV has been highlighted in which the virus will directly transform primary cells; such HCMV strains are named high-risk (HR) HCMV strains. In light of these new findings, this review defines the criteria that characterize HR-HCMV strains and their molecular as well as the phenotypic impact on the infected cell and its tumor microenvironment.

## 1. Introduction

Human cytomegalovirus (HCMV) or human herpesvirus 5 (HHV-5), one of the eight human herpesviruses, can establish lifelong latency within its corresponding host, and possesses the potential for reactivation. Recent investigations have reported the presence of HCMV proteins and DNA in tumoral tissues of glioblastoma, neuroblastoma, colon cancer, breast cancer, ovarian cancer, and prostate adenocarcinoma [1,2,3,4,5], questioning the role of HCMV in the initiation and/or progression of cancer.

So far, seven human oncogenic viruses have been listed: Epstein–Barr virus, (EBV), Kaposi sarcoma human virus (KSHV), human papillomavirus (HPV), hepatitis B and C viruses (HBV, HCV), human T-lymphotropic virus-1 (HTLV-1), and the most recently discovered Merkel cell polyomavirus (MCPyV). Although HCMV is not yet included in the oncogenic viruses list, it becomes more likely that HCMV is the eighth human oncovirus [4,6]. The definitive classification of HCMV as the eighth oncovirus could be greatly facilitated by the discovery of one or several viral oncoproteins (such as E6 and E7 for HPV). In addition, the characterization of strains of oncoviruses directly involved in the transformation process, named high-risk strains (in contrast to non-oncogenic low-risk strains), is of critical importance to unveil the pathophysiology of cancers linked to oncoviruses. The most well-known example is the HPV with high-risk and low-risk strains—the former are at the origin of cervical and head/neck cancers, while the latter are not involved in malignant transformation, but rather in benign tumors such as warts.

HCMV contributes to carcinogenesis as an initiator or promoter [7,8,9]. HCMV allows the tumor to escape immune surveillance by encoding viral proteins and inducing various immunosuppressor cellular factors allowing HCMV-induced immune tolerance, which favors tumor growth. In return, HCMV will increase its fitness in the immunologically weak environment of the cancerous cells [10]. Recently, HCMV has been reported to trigger the generation of polyploid giant cancer cells (PGCCs) as already reported for all human oncoviruses discovered so far [6,11]. PGCCs are keystone cancer cells resulting in stemness, chemoresistance, metastasis, and relapse in poor-prognosis cancers [12]. We and others have suggested revisiting the hallmarks of cancer to add a new hallmark, namely, the PGCCs and giant cell cycling [6,13,14]. In addition, one of the main characteristics of oncoviruses, especially DNA oncoviruses such as HPV and EBV, is that oncogenesis parallels stemness with the appearance of cancer stem cells [15,16,17]. Similar to HPV and EBV, the chronic infection of epithelial cells by some strains of HCMV, named high-risk HCMV, was also characterized by dedifferentiation parallel to the appearance of stemness and cellular transformation [18].

Most of the oncoviruses, especially oncoviruses of the Herpesviridae family, namely, EBV and KSHV, alternate between lytic and latent phases to favor tumor initiation and spread [19]. Similarly, HCMV infection starts with viremia and lytic cycle followed by latency in healthy individuals. Nevertheless, the homeostatic equilibrium established between HCMV and the host could be broken time by time by immunosuppressive conditions, during acute infections (bacterial and viral), transplantation, and even in physiological immune suppression such as in pregnancy. In fact, during a lifetime period, HCMV, through these successive periods of reactivation and latency, will genetically and phenotypically evolve to adapt to a moving immune landscape [20,21,22,23], which could, in some cases, lead to the appearance of cancer stem cells resulting in the existence of “oncogenic” strains.

This review highlights the high variability of the HCMV genome that can impact the HCMV oncogenic potential via newly reported oncogenic signals that contribute to cancers, especially those of poor prognosis, and will lead to the identification of oncogenic high-risk (HR) HCMV strains.

## 2. HCMV from Oncomodulation to Oncogenesis

The prevalence of HCMV is remarkably high in several cancer forms [24]. However, it is very difficult to determine whether the presence of HCMV is incidental due to the viral infection on the top of an already-present tumor in the context of an immunosuppressive tumor microenvironment (TME), or whether HCMV by itself can initiate and promote the cancer program, and thereby could be defined as a genuine oncovirus.

The first paradigm that emerges to explain the role of HCMV in cancer is named oncomodulation, where the virus enhances the oncogenesis process already occurring in the transformed cells. Thus, the oncomodulation paradigm relies on the indirect role of CMV in cancer. HCMV, through its oncomodulatory effect, catalyzes the oncogenic process within the cancerous cells by expressing viral proteins known to possess a pro-oncogenic power such as IE1 and US28, among others, helping tumor cells to evade the immune system, preventing cell death, and favoring cell survival [4]. In addition, the restricted cellular immune responses against HCMV in these immune-privileged tumor sites will enhance HCMV replication in a context of constrained replication as reported previously in the HCMV infection of transformed cells [25]. On the other hand, cancer cells, on their own, escape immune control, thereby favoring epithelial–mesenchymal transition (EMT), metastasis, and relapse. Thus, the combination of the intrinsic cellular machinery and the viral immune escape strategies in cancer cells may offer an environment that enhances limited HCMV replication and boost cancer cells to evade immune surveillance, showing the bidirectional relationship between tumor cells and HCMV [2,10,26].

Besides the oncomodulation paradigm, our research group recently forwarded a new paradigm, namely, a direct oncogenic effect of HCMV [4]. First, among multiple cell types infected by HCMV, the stem cells are permissive to HCMV and stem cells markers such as Thy-1 and platelet-derived growth factor receptor alpha (PDGFRα) favor HCMV infection [27,28,29,30]. Since stem cells, especially cancer stem cells (CSCs), play a critical role in tumor initiation, cellular transformation, tumor heterogeneity, and cancer dissemination through EMT and metastasis, it is worth questioning the direct role of HCMV in oncogenesis potentially through the infection of stem cells and/or the appearance of cancer stem cells. In addition, stem cells can lose control over their self-growth and renewal, act as a cancer source, and become susceptible to oncogenesis in the presence of inflammation and altered DNA repair pathways, the latter of which has been observed frequently with some HCMV strains [31,32]. HCMV could also favor stem cell survival, which would potentially sustain oncogenesis. Thus, IE1 protein promotes the presence of glioblastoma cancer stem cells through the induction of several stemness key markers, namely, SRY-Box Transcription Factor 2 *(*SOX2)*,* Nanog, Nestin, and octamer-binding transcription factor 4 (OCT3/4) [33,34]. In glioblastoma cells, HCMV IE1 protein favored the induction of transcription factors that are crucial for cancer stem cell persistence, cancer growth, and signaling pathways associated with the EMT phenotype [33,35].

In addition to the high diversity of HCMV clinical strains [36,37], a key element to discriminate between the oncogenic and oncomodulatory properties of HCMV strains is the limited potential of HCMV to replicate in already transformed cells [25]. HCMV was unable to productively infect most cancer-derived cell lines due to the fact that oncogenic alleles induce multiple restrictions to HCMV replication [25]. In this experimental model, the strain utilized was BADwt, derived from a bacterial artificial chromosome (BAC) clone of the HCMV AD169 laboratory strain, and the studied cells were fibroblasts [25,38]. In contrast, the clinical HCMV-DB and BL strains isolated in our laboratory displayed oncogenic capacities in primary human epithelial mammary cells (HMECs) and can fully replicate in these cells with the alternation of both lytic and latent viral cycles, resulting in the appearance of CMV-transformed HMECs (CTH cells) up to several months post-infection [11,39,40]. Thus, a specific fitness for long-run epithelial cell replication of the HCMV clinical oncogenic strains has to be taken into account to highlight the oncogenic properties of the virus.

## 3. Link between Oncogenesis, Polyploid Giant Cancer Cells (PGCCs) and High-Risk HCMV (HR-HCMV) Strains

Tumors are renowned as being intricate systems that harbor heterogeneous cancer cells with distinctly diverse molecular signatures, sizes, and genomic contents. Among those various genomic clonal populations within the complex tumoral architecture are polyploid giant cancer cells (PGCCs). Although described for over a century, PGCCs are increasingly being recognized for their prominent role in tumorigenesis, metastasis, therapy resistance, and tumor repopulation after therapy. A shared characteristic among all tumors triggered by oncoviruses is the presence of polyploidy [6]. Recently, our research team highlighted the role of PGCCs as a critical factor following infection with some strains of HCMV which can be described as oncogenic (or high-risk) HCMV strains (in addition to the oncomodulatory effect of the virus) [4]. Two HCMV clinical strains isolated in our laboratory, named HCMV-DB and HCMV-BL, were capable of transforming primary human mammary epithelial cells (HMECs) into cytomegalovirus-transformed HMECs (CTH cells) and producing a “transcriptional profile” associated with DNA hypomethylation that resulted in the appearance of PGCCs in culture parallel to enhanced proliferation, the activation of cancer stem cells, and the EMT process [11,41,42]. In addition, IE1 expression was detected in CTH cells including PGCCs, which also express embryonic stem cell markers [11]. Xenografted NSG mice injected with CTH cells developed tumors that harbor HCMV DNA such as the lncRNA4.9 gene, which displays a triple-negative basal-like phenotype [40]. Thus, following acute infection of HMECs with some HCMV strains (DB, BL), our team showed that transformed cells appear in culture including PGCCs and are tumorigenic in xenografted NSG mice, indicating the direct involvement of HCMV in oncogenesis. The molecular mechanisms involved in HCMV-induced oncogenesis are multifactorial including, among others, cellular stress, polyploidy, and genomic instability parallel to stemness appearance [4,11]. HCMV gene products including IE1 viral protein could affect the pathways of p53 and Rb tumor suppressors, and other pathways that are responsible for DNA repair parallel to the increased expression of c-Myc in the transformed cells [7,43,44]. Presuming the role of HCMV gene products in causing DNA damage directly and indirectly, and stimulating proliferation in stem cells, HCMV (or at least some high-risk HCMV strains) have the potential to initiate and promote tumor formation, especially following acute infection of epithelial cells, which will result in the appearance of adenocarcinoma of poor prognosis such as triple-negative breast cancer (TNBC). In agreement with this hypothesis, we recently isolated two HCMV strains, named B544 and B693, from triple-negative breast tumors, which replicated in MRC5 cells and transformed HMECs toward CTH cells after acute infection [18,45]. Altogether, our data indicated that the oncogenic potency of HCMV clinical strains varies between low- and high-risk strains [11,39,40,42], further opening the door to their in-depth characterization.

## 4. High-Risk versus Low-Risk HCMV

**Figure 1:**
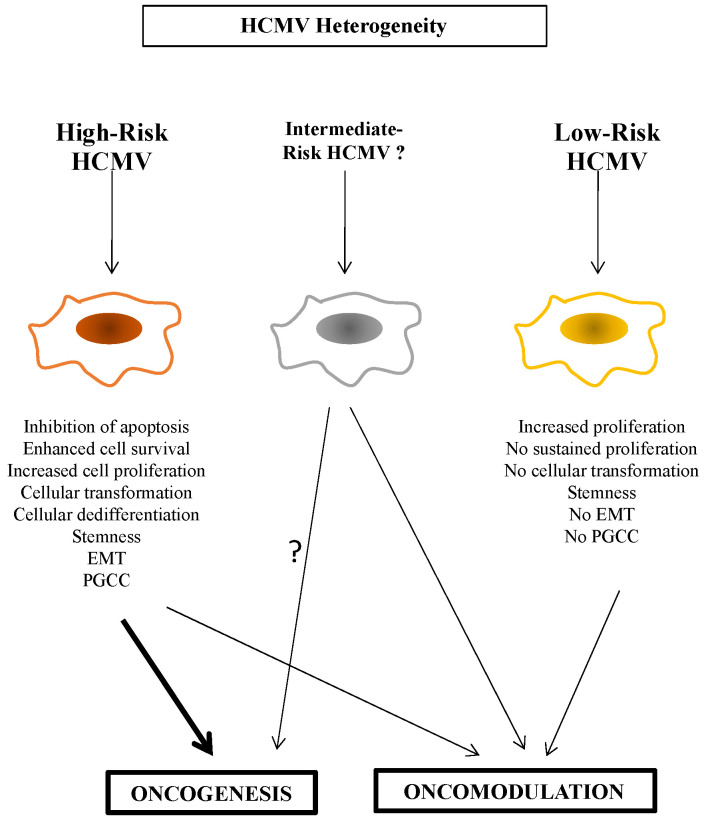
Oncomodulation, oncogenesis, and HR/LR HCMV strains.

**Figure 2:**
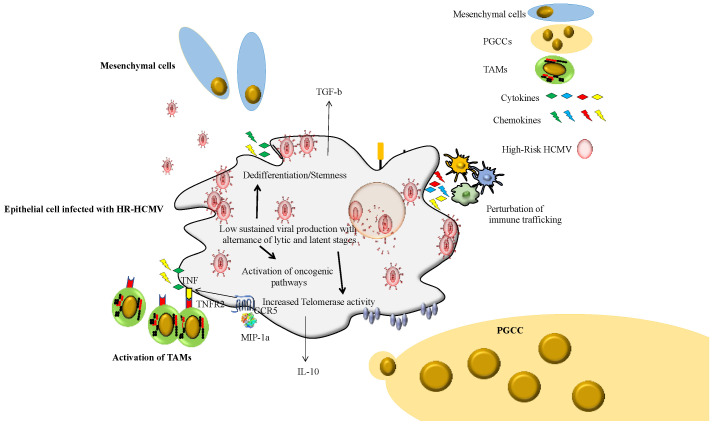
Impact of the HR-HCMV strains in the infected cell landscape and tumor microenvironment.

To further discriminate between high-risk (HR) and low-risk (LR) HCMV strains, it is critical to characterize the viral strain involved, the biological traits of the infected cell, and its cellular microenvironment, in vitro as well as in vivo. For example, only the HR-HCMV strains can trigger the appearance of PGCCs (Figure 1) [4,46]. HCMV-DB and HCMV-BL have been classified as HR strains since they showed a sustained transformation of acutely infected HMECs in vitro. Similarly, the two HCMV strains isolated from TNBC biopsies, HCMV-B544 and -B693, transformed HMECs toward CTH cells and PGCCs in vitro [18,45]. At the molecular levels, these HR strains were characterized by elevated Myc expression, PI3K/Akt pathway activation, and p53 and Rb gene repression [11,40,47,48]. With regard to immune responses, Myc suppresses immune surveillance by modulating the expression of the innate immune regulator (CD47, also known as integrin-associated protein), the Treg activity, and the adaptive immune checkpoint, namely, programmed death ligand 1 (PD-L1) [49]. Further, PI3K/Akt hyperactivation observed with HR-HCMV strains [11,40] induces immunosuppression and favors tumor initiation and progression via the activated Notch pathway [50]. The loss of Rb leads to an increase in IL-6 production recently involved in the appearance of PGCCs in ovarian cancer [51,52]. We also observed the high expression of the EZH2 methyltransferase belonging to the PCR2 complex parallel to enhanced Myc expression in HMECs chronically infected with the two HR-HCMV strains B544 and B693 in vitro; both EZH2 and Myc markers were also enhanced in TNBC biopsies tissue harboring these two strains [18,45]. In contrast, the LR-HCMV strains did not favor the sustained expression of Myc, Akt, or EZH2 following acute infection of HMECs resulting in cell death in chronically infected cultures [11]. In agreement with these in vitro data, we detected clinical HCMV strains in breast cancer biopsies mostly in luminal breast cancer, but also in some TNBC, with no increase of “oncogenic” markers such as Myc, EZH2, Akt, and Ki67Ag in tumor tissue [11], and should therefore be referred as LR-HCMV strains. Altogether, in contrast to LR-HCMV strains, HR-HCMV strains trigger the enhanced expression of cellular markers actively involved in oncogenesis such as Myc, Akt, and EZH2, especially in cancers with poor prognosis (Figure 2).

Although HR-HCMV strains are potentially involved in the oncogenesis process as described previously [4,11,18,40,45], they favor dedifferentiation and stemness during the transformation process. Stemness markers such as Nanog, SOX2, OCT4, and SSEA4 were highly expressed in HMECs chronically infected with HR strains in vitro, parallel to the downregulation of differentiation markers such as EpCAM [11]. Parallel to dedifferentiation and stemness, total and partial EMT were observed in HMECs chronically infected with HR-HCMV [11,53]. The co-expression of E-cadherin and vimentin corresponds to partial EMT wherein intermediate hybrid epithelial and mesenchymal phenotypes coexist, ensuring the plasticity of these cells [54] while maintaining similar tumor-propagating cell capacity [55,56]. Altogether, dedifferentiation, stemness, and EMT accompany the oncogenic process triggered by HR-HCMV and lead to the appearance of cancers of poor prognosis such as triple-negative breast cancer [11,18,45]. We cannot dismiss that HR-HCMV strains also favor the appearance of high-grade glioma such as glioblastoma, high-grade serous ovarian cancer (HGSOC), and prostate cancer with metastasis, relapse, and therapy resistance.

In contrast, the LR-HCMV strains identified so far only resulted in “opportunistic infections” with no or very limited impact on the cellular microenvironment and did not trigger oncogenesis. Nevertheless, we cannot underestimate the role of LR-HCMV strains in oncomodulation where they could favor the rapid progression of already transformed cells. This could occur through at least two distinct mechanisms. First, the LR strains could activate oncogenic pathways already at play in the transformed cells, e.g., IL6-STAT3, AKT, or Myc axes [57]. Second, they could accelerate oncogenesis through an indirect mechanism of immune suppression that will be mostly tested in vivo. Although HR-HCMV strains can trigger oncogenesis, their additional role as oncomodulatory factors cannot be excluded so far (Figure 1).

HCMV infection fulfills all of the hallmarks of cancer, including sustained proliferative signaling, deregulating cellular energetics, resisting cell death, favoring genome instability and mutation, inducing angiogenesis, activating invasion and metastasis, tumor-promoting inflammation, enabling replicative immortality, and avoiding immune destruction [53]. Although various HCMV strains might fulfill some or all of the hallmarks of cancer, HR-HCMV strains could specifically activate invasion and metastasis, evade growth suppressors and contact inhibition, favor sustained dedifferentiation, stemness, PGCC formation, and oncogenesis in tumors of poor prognosis (Table 1).

## 5. Characterization of High-Risk HCMV Strains

To nail down the direct role of HR-HCMV strains in cancer, it will be critical to isolate and characterize additional HR-HCMV strains from patients. So far, we have isolated four HR-HCMV strains either from biological fluids (urine, bronchoalveolar lavage) or directly from tumor biopsies. Two clinical HR-HCMV strains (DB and BL) were isolated from biological fluids among 20 tested patients; two clinical HR-HCMV strains (B544 and B693) were obtained from tumor biopsies among 19 tested BC patients. Thus, an average of around 10% of the HCMV strains isolated so far in our laboratory were considered HR-HCMV strains.

We also observed that all four HR-HCMV strains isolated by our group replicated very slowly in MRC5 cultures of fetal lung fibroblast cells with an average detection in culture by CPE and IF of around 20 days post-infection. In contrast, some LR strains replicated very quickly in the MRC5 cultures. Thus, the fitness of HR-HCMV strains for the MRC5 culture is low, which makes it difficult to isolate in the medical laboratory. This will have to be confirmed in future studies. It could also explain why the HR-HCMV strains often require several months post-infection to transform the epithelial cells. We also observed an alternation of the lytic and latent stages in the chronically infected cultures with HR strains that will ultimately result in cell transformation after several months in culture [40].

The cellular tropism of the HR-HCMV strains also needs to be tested further. Although we observed a poor tropism of HR-HCMV strains for MRC5 cells and a limited tropism for epithelial cells with lytic/latent phases, the tropism of HR-HCMV strains for monocytes/macrophages needs further investigation. In fact, the first HR-HCMV strain isolated by our group, the HR-HCMV-DB strain, displayed a very high tropism for primary macrophages (monocyte-derived macrophages, MDMs) in vitro [58]. HCMV-DB infection of MDMs increased p52 binding activity without activating the canonical p50/p65 complex. Moreover, Bcl-3 was upregulated and demonstrated to associate with p52, indicating p52/Bcl-3 complexes as the major component of the NF-kappaB complex in MDMs. Luciferase assays in promonocytic U937 cells transfected with a major immediate early promoter (MIEP)-luciferase reporter construct demonstrated MIEP activation in response to p52 and Bcl-3 overexpression. A chromatin immunoprecipitation assay demonstrated that p52 and Bcl-3 bind the MIEP in acutely HCMV-infected MDMs. Thus, the activation of p52/Bcl-3 complexes in MDMs in response to HCMV-DB infection resulted in the activation of the pro-oncogenic Bcl3 factor parallel to an M2 phenotype. Interestingly, the tumor-associated macrophages (TAMs) display an M2 phenotype with TGF-beta secretion, a critical factor in EMT and progression of cancers with poor prognosis such as TNBC and glioblastoma [59,60]. Definitively, the phenotype and function (TAM-like) of HCMV strains, especially HR-HCMV strains, should be assessed in monocytes/macrophages, a critical cell type in the tumor microenvironment, but also a critical site of virus latency [61] (Figure 2). The tropism of primary HCMV strains should also be tested in endothelial cells, since vascular tumor invasion is a factor of poor prognosis. We observed that HR-HCMV strains were isolated from TNBC biopsies with vascular emboli.

Following the isolation of HR-HCMV strains based on cellular transformation both in vitro and in vivo, it is of critical importance to study the genome of the HR-HCMV strains and to compare it with LR-HCMV strains in order to determine whether some genomic motif(s)/domain(s) are conserved among HR-HCMV strains and are distinct in LR-HCMV strains. For example, the E6 genes of HPV-16 and HPV-18 display a PDZ-binding motif (X-T/S-X-V) that is a specific genomic signature for the HR-HPV strains [62]. Whole-genome sequencing of HCMV could be one of the best tools to compare the sequences of all HCMV genes and check whether one or several viral genes with specific “HR-motif(s)/domain(s)” could be detected. We already sequenced the genome of the HR-HCMV strains DB and BL with Davison’s group (Genbank KT959235 and MW980585, respectively) [11,23,40]. Various techniques, including restriction fragment length polymorphism (RLFP) analysis [63], targeted amplicon sequencing [64,65,66,67], and whole-genome sequencing [21,68], have been used to describe HCMV genome variability. With the increasing sequencing depth of next-generation sequencing platforms, the detection of low-frequency variants, i.e., minors, has become possible [69]. Currently, there is mounting evidence that HCMV exists as a heterogeneous collection of genomes with variations in composition and distribution between anatomical compartments [70,71] and over time [21,68,69,71]. Performing high-throughput analysis, HCMV genome diversity is significantly more divergent than all other human herpesviruses and highlights the capacity of the viral genome to adapt to its host environment with high flexibility, and maybe in some cases to become oncogenic [72]. Altogether, a systematic analysis of HCMV strains, especially those present in tumor biopsies, will be critical to unveil the HR strains and define the genomic motifs involved in transformation.

## 6. Open Questions on HR-HCMV Strains

Deciphering the role of HR-HCMV strains versus LR-HCMV strains requires addressing several points. First, as stated above, the frequency and molecular profile of the HR-HCMV strains in human diseases, especially cancers, have to be clarified. Second, the oncoviruses are usually necessary, but not sufficient, to trigger cancer; therefore, additional factors in addition to the HR-HCMV strains should be at play and further characterized. Third, the oncoviruses, especially the DNA oncoviruses, usually favor several cancers. For example, HR-HPV can cause cervical cancer, but also penile cancer, anal cancer, and cancers of the mouth and throat. Similarly, EBV favors Burkitt’s lymphoma, nasopharyngeal carcinoma, as well as Hodgkin’s and non-Hodgkin’s lymphomas [73,74]. KSHV triggers Kaposi sarcoma, but also a form of multicentric Castleman disease (KSHV-MCD) and primary effusion lymphoma (PEL) [75]. Therefore, the HR-HCMV strains could favor oncogenesis not only in breast cancer, but also in other adenocarcinomas (derived from transformed epithelial cells) such as prostate, colon, or ovarian cancers. Since HCMV infects glial cells, we cannot exclude the direct involvement of HR-HCMV strains in the appearance of glioblastoma. Fourth, the presence of episomal and/or integrated forms of HR-HCMV genome in transformed cells is definitively an important question to answer, since viral genome integration could be mostly observed with HR-HCMV strains, as already reported for HR-HPV [76,77]. Fifth, as stated above, the identification of the HCMV gene(s) involved in the transformation induced by the HR strains is considered essential, since it will definitively confirm that HCMV is the eighth human oncovirus. Finally, the use of animal models to study, in vivo, the oncogenic potential of cytomegalovirus strains is of critical importance [78]. In the future, clinical studies will certainly be performed to link the presence of HR-HCMV strains to cancer development, especially in tumors of poor prognosis.

## 7. Conclusions

For decades, HCMV has been considered a herpesvirus involved mostly in asymptomatic or mild disease in immunocompetent individuals, which could lead to an important burden of disease during congenital infection and in immunocompromised patients. Recently, the long-lasting effects of HCMV started to arise in cancer. The oncomodulation paradigm could explain the accelerated progression of cancers when HCMV superinfection of the tumor occurs. Recently, a direct oncogenic effect of some HCMV strains, named HR-HCMV strains, has been observed with cellular stress, PGCCs, stemness, and EMT, which could explain the appearance of aggressive cancers, especially adenocarcinoma with poor prognosis, metastasis, and resistance to treatment. A better characterization of HR-HCMV strains, similar to HR-HPV, will highlight the molecular mechanisms involved in cellular transformation and sustained oncogenesis in addition to emphasizing the characteristics of the tumor microenvironment in the presence of the virus. This could result in better diagnostic tools (genotyping) and ultimately pave the way to new therapeutic approaches including anti-HCMV treatment, immunotherapy, and prophylactic vaccines.

## Figures and Tables

**Table 1:** Characteristics of HR and LR HCMV strains.

	High-Risk HCMV	Low-Risk HCMV
**Virological characteristics**		
• Isolation from biological fluids *	+++	+++
• Isolation from tumor biopsies	+++	+++
• Growth in MRC5 cultures	+	+++
• Long-term growth in epithelial cells	+++	−
**Molecular Pathways**		
• Activation of Myc	*+++*	*+*
• Suppression of Rb and p53 activity	*+++*	*+*
• Activation of PI3K/AKT pathway	*+++*	*+*
• Activation of STAT3 pathway	*+++*	*+*
• Activation of telomerase	*+++*	*+/−*
**Cellular Effects**		
• Inhibition of apoptosis	+++	+/−
• Enhanced cell survival	+++	+/−
• Increased proliferation	+++	++
• Cellular transformation	+++	−
• Cellular dedifferentiation	+++	+/−
• Stemness	+++	+
• EMT	+++	−
• PGCC appearance	+++	−
**In vivo**		
• Tumor in xenografted mice	++	−
• Detection in human tumor biopsies	++	*++*
• High-risk human tumor landscape **	+++	*+*

* Urine, bronchoalveolar lavage; ** tumor with high expression of Myc, Ki67, and EZH2.
